# Supplementary material for: Are carbon emissions trading and green financial instruments synergistic? -Comprehensive quantitative research based on content analysis
Source: PLoS One. 2024 Mar 7;19(3):e0298601. doi: 10.1371/journal.pone.0298601 (PMC10919601; doi:10.1371/journal.pone.0298601)
Supplement: S1 Data — (ZIP) [file pone.0298601.s001.zip › Data/地级市Z_F工作报告.zip!/─┌├╔╣┼/║⌠┬╫▒┤╢√╩╨/└┤╫╘╣1⁄2╓┌║┼í╛┬φ┐╦╩2╛▌═°í┐_2019.docx]

各位代表：

现在，我代表呼伦贝尔市人民政府向大会报告工作，请予审议，并请市政协委员和列席会议的同志提出意见。

一、2018年工作回顾

过去的一年，在自治区党委、政府和市委的坚强领导下，市政府团结带领全市各族人民，以习近平新时代中国特色社会主义思想为指导，深入贯彻党的十九大，十九届二中、三中全会精神和习近平总书记对内蒙古重要讲话重要指示批示精神，牢牢把握稳中求进工作总基调，坚持生态保护优先、走好绿色发展之路，统筹做好稳增长、促改革、调结构、惠民生、防风险各项工作，保持了经济平稳发展、社会繁荣进步、民族团结奋进的良好局面。

（一）综合实力稳步增强。主要经济指标实现平稳增长。地区生产总值完成1253亿元，增长3%。一般公共预算收入完成86亿元，增长0.9%。一般公共预算支出完成449亿元，增长3.2%。城乡常住居民人均可支配收入完成33401元和14895元，分别增长7.1%和9.7%。农牧业再获丰收，粮食产量达140亿斤。优质农畜林产品进入北京超市，钓鱼台·呼伦贝尔美食节、北京平谷呼伦贝尔绿色优质农畜产品推介会成功举办，全国奶业振兴工作推进会和奶业D20峰会成功承办，“中国芥花油之都”成功命名，新增国家级重点龙头企业1家，“三品一标”认证总数达到326个，秀水乡大米荣获第十九届中国绿色食品博览会金奖，呼伦贝尔绿色食品知名度、美誉度进一步提升。新旧动能转换接续逐步加快，规模以上工业总产值完成533.9亿元，云计算、大数据、生物科技、临空产业、生物质发电等新兴产业开始破题，工业经济正在形成新的基础。商贸物流、健康养老、电子商务等现代服务业加快发展，社会消费品零售总额完成679亿元，增长6.2%。旅游业发展势头强劲，全年接待游客1865万人次，增长8.4%，旅游收入685亿元，增长12.8%。呼伦贝尔大草原、额尔古纳湿地5A级景区创建步伐加快。扎实开展景区景点清理整顿工作，处置整顿违规景区景点402个。森林草原旅游列车项目稳步推进。“冰雪那达慕”“冬季英雄会”“中俄蒙国际冰雪节”“中国冷极节”等系列活动做热呼伦贝尔冬季游。

（二）质量效益同步提升。结构变化稳步向好，服务业增加值增速5.1%，占地区生产总值的49.3%。先行性指标保持较快增长，全社会用电量增长9.7%，工业用电量增长6.7%，铁路货运量增长9.2%。全年新增市场主体2.7万户，内资企业户数增长9.9%。绿色发展指标体系全面实施，污水集中处理率达到95%，草原综合植被覆盖度明显提高。一批推动传统产业升级、促进新兴产业发展的重点项目加速推进，陈旗草产业集聚区、阿荣旗肉牛繁育基地等项目开工建设，额尔古纳塞尚雀巢新产品生产线加快建设，阜丰、荣达、华德、蒙拓等企业智能化升级步伐加快，晟通糖业投产运营；生物质热电联产项目推进较快，海国华创大数据中心开工建设，国信优易大数据创新创业基地签约落地，齐鲁制药项目前期工作进展顺利，中国民航大学内蒙古飞行学院挂牌运行。园区振兴全面推进，完成经济技术开发区总体规划修编，现代农牧业产业园开工建设，岭东绿色有机食品产业园启动规划。

（三）三大攻坚战初战告捷。制定出台债务化解、脱贫攻坚、污染防治等方面的行动计划、实施方案和具体措施，坚持高位推动，强化工作调度，务求攻坚实效。全年化解政府性债务100.1亿元，超额完成年度任务；牧业四旗治理民间高利贷工作取得重大进展，化解高利贷存量1.2亿元。突出“精准”二字，狠抓问题整改，重点落实产业帮扶、易地搬迁、生态补偿、发展教育、就业扶持、健康扶持、社会保障兜底等措施，实施京蒙对口帮扶项目67个，各级财政投入扶贫资金10.9亿元，实现14897人脱贫、67个贫困嘎查村出列，4个自治区级贫困旗市完成摘帽退出评估验收，贫困发生率降至0.9%。全面打响“蓝天、碧水、净土”保卫战，认真抓好中央环保督察及“回头看”反馈问题整改，呼伦贝尔中心城区及59个农村牧区饮用水水源地保护、12座城镇污水处理厂提标改造等6项年度整改任务基本完成；全面开展生态环境突出问题排查治理“百日攻坚”行动，查处案件259件；全面启动第二次全国污染源普查和土壤污染状况详查。

（四）生态建设成效显著。坚持保护、治理并重，坚守生态红线、环境底线。加快推进呼伦湖生态综合治理，一期工程18个项目全部建成，呼伦湖整改要求落到实处；二期工程启动实施，呼伦湖水量稳定增加、水质逐步改善。全面实施呼伦贝尔大草原、大兴安岭森林、河湖水系、黑土地保护治理工程。认真落实新一轮草原生态保护补助奖励政策，发放补奖资金6.27亿元，退牧还草145万亩，人工种草350万亩，禁牧1687万亩，草畜平衡8671万亩。扎实推进草牧业试验试点区建设。巩固天然林商业性停伐和退耕还林成果，持续推进天然林保护、森林生态效益补偿、三北防护林、森林质量提升等重点工程，完成营造林251万亩、新造林52.52万亩，清退已垦林地26万亩。河长制、湖长制深入落实。呼伦贝尔水生态文明试点城市建设通过国家验收。继续实施沙区综合治理，建立草原沙化监测系统。扎实开展“大棚房”清理整治工作。制定生态文明建设目标体系和评价考核办法，完成生态保护红线划定基础工作。

（五）发展条件持续改善。坚持城乡统筹，加快基础设施建设，保障能力不断提高。实施市政基础设施项目216个，房地产开工802万平方米，各类保障性安居工程开工18372套、建成18148套，完成农村牧区危房改造1400户。阿荣旗—扎兰屯铁路货运通车。海拉尔—满洲里高速公路开工，扎兰屯—阿荣旗等3条公路通车，新改建农村公路400公里。海拉尔机场新航站楼投入使用，满归机场主体工程完工。阿伦河、诺敏河等5个重要支流治理工程进展较快，尼尔基水利枢纽下游灌区项目主体工程完工。110千伏及以上电网工程建设项目有序推进，岭东—冯屯500千伏输变电工程全线竣工。启动“气化呼伦贝尔”工程，中俄东线天然气管道呼伦贝尔支线工程积极推进。

（六）改革开放步伐加快。完成经济、生态及社会事业领域改革任务48项。“放管服”改革不断深化，出台行政审批服务“马上办网上办一次办”事项目录，在全区率先试点“证照分离”和企业名称自主申报改革，实现企业设立登记全程电子化和外商投资企业“一口办理”，企业工商登记时间压缩到3个工作日以内，开办时间压缩到5个工作日以内，不动产登记“一窗受理”改革走在全区前列。农垦改革、国有林场改革任务基本完成。持续推进电力直供交易，降低企业成本5495万元。出台推进企业上市挂牌三年实施计划，成立农牧业融资担保有限公司，积极支持民营经济发展，民营经济总量、就业、税收占比分别达到46.5%、52.2%和51.9%。出台《呼伦贝尔关于口岸建设发展的指导意见》等文件，加快推进中俄蒙合作先导区建设，黑山头互市贸易区正式运营，额布都格互市贸易区通过验收，边民互市贸易政策正在向全市覆盖。海关总署批准设立额布都格海关和阿日哈沙特海关，两个口岸即将通过常年开放验收。电子口岸视频指挥系统建设完成。满洲里市获批国家首批边境旅游试验区。全市口岸进出境货运量完成3268万吨，外贸进出口总额完成189亿元。成功举办海拉尔第十四届中俄蒙经贸展洽会，签约额76亿元，现场交易额1.8亿元。成功举办北京“绿色发展·合作共赢”招商引资推介会。

（七）社会建设全面进步。坚持把保障和改善民生作为出发点和落脚点，人民群众获得感、幸福感明显增强。民生领域支出336.2亿元，占一般公共预算支出的75%。就业形势持续向好，城镇新增就业31453人，农牧民转移就业62052人，高校毕业生就业11325人，零就业家庭至少1人就业。支出就业补助资金1.5亿元，发放创业担保贷款4677万元，创业园、孵化基地达58家，入驻企业3000余户。社会保障更加有力，企业退休职工基本养老金每月提高135元，城乡居民医保财政补助每年提高40元，城市最低生活保障标准月提高40元，农村牧区最低生活保障标准年提高1220元，失业保险金上调10%。社会事业蓬勃发展，提前完成国家义务教育发展基本均衡县创建任务。呼伦贝尔市农业科学研究所参与的“大豆优异种质挖掘、创新与利用”项目荣获国家科学技术进步奖二等奖。成立呼伦贝尔市直属乌兰牧骑，公共文化标准化均等化建设取得积极进展。医药卫生体制改革稳步推进，医疗服务水平不断提升。我市在全区第十四届运动会上获金牌、奖牌、积分三项第一，第十四届全国冬运会主场馆一期工程试运行，二期主体工程完工，各项筹备工作稳步推进。完成首轮全国文明城市测评。积极推进国家卫生城市创建。我市被命名为“全国民族团结进步创建示范市”。成功举办莫旗、鄂温克旗成立60周年庆祝活动。编纂鄂伦春语、鄂温克语和达斡尔语常用词词典。安全生产形势稳定，各类事故死亡人数下降49％。涉稳矛盾隐患排查有序开展，化解信访积案446件。深入开展扫黑除恶专项斗争，治安防控体系建设水平显著提升，平安呼伦贝尔建设取得新成效。人事人才、国资监管、食品药品、档案史志、审计、统计、气象、人防、地震、国防动员、双拥、边防、老龄、工青妇、红十字、关心下一代等工作均有新进展。

（八）政府自身建设明显加强。切实提高政治站位，树牢“四个意识”，坚定“四个自信”，坚决做到“两个维护”，确保中央各项决策部署落到实处。坚持依法行政、高效施政、从严治政，严格执行“三重一大”决策程序，行政决策民主化、科学化、法治化水平不断提高。自觉接受人大法律监督和政协民主监督，认真听取各民主党派、工商联和无党派人士意见，办理人大代表议案1件、建议151件，政协委员提案268件，办复率100%。严格落实管党治党主体责任和“一岗双责”，持续加强政风建设，政府服务能力、办事效率、队伍形象进一步提升。

各位代表，这些成绩的取得，是自治区党委、政府和市委坚强领导的结果；是市人大、政协监督支持的结果；是全市各族人民奋力拼搏的结果；是各民主党派、人民团体、驻呼部队和社会各界广泛参与的结果。在此，我代表市人民政府向全市各族人民，向所有关心、支持和参与呼伦贝尔发展的同志们、朋友们，表示衷心的感谢!

回顾一年来的工作，我们深刻体会到：履行好政府职能职责，推进经济社会平稳健康发展，必须始终坚持以习近平新时代中国特色社会主义思想为指导，深入贯彻新发展理念，坚持生态保护优先、走好绿色发展之路，切实筑牢祖国北方生态安全屏障；必须始终坚持以人民为中心的发展思想，更好满足各族群众的新需求，不断提高人民群众的获得感、幸福感、安全感；必须始终坚持守望相助、团结奋斗，不断巩固民族团结、和谐安宁的良好局面，切实筑牢祖国北疆安全稳定屏障；必须始终坚持“干”字当先，压实责任，勇于任事，敢于担当，主动作为，抓重点、补短板、强弱项、攻难点，以重点突破带动全面发展。

在看到成绩的同时，我们必须正视发展中存在的困难和问题，主要是：经济运行稳中有变、稳中有忧，外部环境复杂严峻，经济下行压力仍然较大；发展质量不高，传统动能升级缓慢，新动能尚处于培育阶段，经济发展内生动力不足；立市、强市、富市项目不多，资源要素组合力度还需加大，项目储备和项目谋划亟待加强；各级财政特别是旗市区财政保障能力不足，收支矛盾突出，化解债务、防范金融风险压力较大；城乡之间和地区之间发展不平衡，基础设施与公共服务仍有欠账；政府职能转变还不到位，营商环境亟待进一步优化，部分干部谋划工作视野不宽、主动性不强，思想观念、思维方式、能力水平与高质量发展要求还不相适应，等等。对此，我们将坚持问题导向，认真加以解决。

二、2019年工作安排

今年是新中国成立70周年，是全面建成小康社会关键之年。当前，我市面临许多可以抓住用好的重大机遇，国家和自治区明确较大幅度增加地方政府专项债规模，加快实施一批补短板、增后劲的重点项目，大力推进东北全面振兴，继续加大西部大开发力度，并且在全力支持实体经济发展、促进民营企业发展、深入推进改革开放等方面出台了一系列政策措施。同时，我们坚决挤水分、去产能，积极抓改革、促创新，努力调结构、强基础，卸掉了沉重包袱，破除了许多障碍，积蓄了发展动能。只要我们坚定发展信心，保持战略定力，发挥比较优势，团结一心，苦干实干，就一定能够开创高质量发展的新局面。

今年政府工作的总体要求是：以习近平新时代中国特色社会主义思想为指导，全面贯彻党的十九大，十九届二中、三中全会和中央经济工作会议精神，深入落实自治区党委十届八次全会、市委四届九次全会暨经济工作会议精神，统筹推进“五位一体”总体布局，协调推进“四个全面”战略布局，坚持稳中求进工作总基调，坚持新发展理念，坚持推动高质量发展，坚持生态保护优先、走好绿色发展之路，全面贯彻“巩固、增强、提升、畅通”八字方针，以“项目建设推进年”为抓手，以打好三大攻坚战为重点，以保障和改善民生为根本，紧紧围绕“两区三地一家园”目标，统筹推进稳增长、促改革、调结构、惠民生、防风险各项工作，奋力开创美丽富饶和谐安宁呼伦贝尔建设新局面，为全面建成小康社会收官打下决定性基础，以优异成绩庆祝中华人民共和国成立70周年。

经济社会发展主要预期目标：地区生产总值增长3%左右；社会消费品零售总额增长6.5%左右；城乡常住居民人均可支配收入分别增长7%左右和8%左右；保障性民生支出稳定增长；约束性指标达到自治区要求。绿色发展体系主要预期目标：草原综合植被覆盖度稳定在70%以上；森林蓄积量稳定在12亿立方米以上；污水集中处理率达到96.5%。

为实现上述目标，重点抓好以下十个方面工作。

（一）坚持抓重点、攻难点，坚决打好三大攻坚战。继续把三大攻坚战作为政治考验和能力检验，下更大决心，采取更有力措施，针对突出问题，坚决打好重点战役。

打好防范化解重大风险攻坚战。今年要确保完成化解政府隐性债务45.2亿元，确保完成与“十个全覆盖”工程直接相关项目拖欠工程款化解任务。按照“谁举债、谁偿还、谁负责”原则，压实责任，创新方法，综合运用增加化解财力、争取债券资金、清理专项资金、盘活国有资产、压缩一般性支出、债权资本化、审价核减和运用金融融资平台等措施，开前门，堵后门，全力解决政府隐性债务较高问题。强化财政约束，严格规范地方政府举债融资行为，严格政府投资项目审批和管理，从严控制政府债务增量。压缩一般性财政支出，除刚性支出和重点项目外，其他项目支出、“三公”经费等一般性支出总体压缩10%以上，沉淀两年以上的资金一律收回。继续防范和处置非法集资，综合治理民间高利贷等违法违规金融活动。推进融资平台公司转型发展，引导金融服务高质量发展。

打好精准脱贫攻坚战。坚持“两不愁、三保障”标准，将“精准、可持续、激发群众内生动力和提升自我发展能力”三个关键要求贯穿脱贫攻坚全过程，确保7000人减贫、39个贫困村退出、2个国贫旗摘帽。坚决完成中央脱贫攻坚专项巡视反馈意见和2018年度扶贫成效考核反馈问题整改任务。抓好统筹规划，做好脱贫攻坚与乡村振兴战略统筹衔接。深入实施产业扶贫，大力发展贫困人口受益的现代农牧业、劳动密集型加工业、乡村文化旅游和电商服务业，着力构建“户有致富产业、村有特色产业、乡有支柱产业、旗有龙头产业”的扶贫产业发展格局，不断增强贫困地区、贫困群众内生动力和自我发展能力。实施脱贫攻坚“清零达标”专项行动，全面摸清底数，逐条推进落实。抓好扶贫资金绩效管理，整合各类扶贫资金，加快项目实施，确保扶贫资金规范使用。深化京蒙协作和定点帮扶，抓好项目对接落地。巩固脱贫攻坚成果，健全完善稳定脱贫机制，确保脱贫结果真实、可持续。

打好污染防治攻坚战。深入实施污染防治三年行动计划，坚决完成中央环保督察及“回头看”、草原生态环境专项督察反馈问题和“百日攻坚”发现问题整改年度任务。打好蓝天保卫战，完成60万千瓦燃煤发电机组改造和旗市区政府所在地建成区10蒸吨及以下燃煤锅炉年度拆改任务，做好供热燃煤锅炉“冬病夏治”、燃煤电厂超低排放、柴油货车污染治理等工作。打好碧水保卫战，落实最严格水资源管理“三条红线”指标，大力实施安全饮水工程，深入开展集中式饮用水水源地环境保护专项行动，完成10个城镇水源地规范化建设；强化涉水行业企业环境监管，提升污水收集处理能力和再生水利用率。打好净土保卫战，组织开展土壤污染状况详查、重点行业企业用地调查和定期监测，做好生活垃圾分类管理和处置工作，加强农村牧区面源污染防治。

（二）坚持抓治理、强监管，切实提高生态文明建设水平。全面贯彻习近平生态文明思想，坚决落实总书记关于呼伦湖综合治理重要指示批示精神，统筹山水林田湖草系统治理，切实守护好蓝天绿地、碧水青山。

加大生态保护建设力度。紧紧围绕“水质改善、水量增加、生态修复”的目标，做好呼伦湖生态环境综合治理，扎实推进二期投资17.8亿元的13个项目，确保完成年度任务；加快实施呼伦湖周边及流域城镇生活污水处理厂再生水利用工程；积极推动《呼伦湖流域生态与环境综合治理实施方案》阶段性评估与修编；加快推进呼伦湖水环境保护形势与水质改善研究；加强呼伦湖科研监测体系建设，提高综合管理水平。严格落实新一轮草原生态保护补助奖励政策，完成禁牧和草畜平衡任务，退耕还草8万亩，退耕还林12万亩。实施“三北”防护林、天然林保护等工程，加大大兴安岭北部原始林区保护力度。争取将已垦林地草原纳入国家退耕还林还草政策支持范围。积极争取国土空间综合整治生态修复项目。继续推进草牧业试验试点区建设，支持呼伦贝尔农垦与中科院合作申报国家级草牧业重点工程实验室项目。

加强生态文明制度建设。建立严格的生态管控体系，实现一条红线管控重要生态空间。强化生态保护红线、环境质量底线、资源利用上线和环境准入负面清单管理。落实主体功能区规划，推进“多规合一”，加快实现国土空间管控一张蓝图。制定生态环境损害赔偿、资源有偿使用和生态补偿制度。抓好自然资源资产负债表试点和领导干部自然资源资产离任审计，完善生态环境损害责任终身追究制。健全河湖长制配套制度。完善生态环境监管体制，严厉打击破坏生态环境行为，用最严格的监管制度守住生态建设成果。积极创建国家级生态文明建设示范市。举办好首届绿色发展大会、绿色产业博览会。加强生态文明教育，让绿色生活成为时尚，让保护环境成为习惯。

（三）坚持强责任、抓项目，不断扩大投资需求。深入实施“项目建设推进年”，将项目建设作为撬动经济发展的总开关，以项目补短板、增优势、强支撑，以高质量项目促进高质量发展。今年计划实施市级重点项目220个，总投资832亿元，年度计划投资283亿元。

抢抓进度促开工。全力推动项目开工建设，加快推进土地、规划、环评、能评、融资、招标等前期工作，制定项目建设时间表、路线图，倒排工期，加快进度，重点项目开复工率要达到90%以上，力保全市固定资产投资实现正增长。

凝心聚力谋储备。围绕市、旗两级发展定位、产业基础、优势特色，用足、用活、用好国家支持民族地区、边疆地区、重点生态功能区加快发展系列政策，统筹抓好项目谋划，高质量包装项目，分类分批建设项目库，加快形成“开工一批、建设一批、储备一批、谋划一批”的滚动发展格局。

精准施策优服务。积极构建“亲”“清”新型政商关系，为项目建设提供优质、高效、便捷服务。全力抓好经济运行和重点项目日常调度，积极主动为企业做好服务，加强要素保障，履行招商承诺，坚决打通“中梗阻”，做到全程沟通、全程协调，为项目建设和企业发展营造良好环境。

狠抓落实强责任。实化细化重点项目包联工作机制，进一步压实各地区各部门抓项目的主体责任，明确目标任务，加大项目统筹调度、督查通报、问效问责力度，建立项目工作激励机制。加大招商引资和项目争跑力度，建设招商引资信息平台，创新招商引资方式方法，在全市形成抓招商、跑资金、上项目的浓厚氛围。

（四）坚持兴产业、创示范，深入实施乡村振兴战略。准确把握乡村振兴科学内涵，坚持规划先行、示范引领、统筹推进，确保取得实效。

全力以赴抓基地建设。加快发展种植业，走稳粮扩饲、种养加结合之路，玉米种植稳定在500万亩左右，大豆种植稳定在1000万亩左右，建设大豆良种繁育基地2万亩，黑土地保护利用90万亩，耕地轮作130万亩，新增农业生产标准化面积240万亩。加快发展养殖业，以奶牛、肉牛、肉羊为重点，发展规模化、集约化养殖。加快优良品种培育，推进草原短尾羊通过国家品种审定。抓好三河牛选育、扩繁，年内新增三河牛1万头，打造三河牛繁育养殖核心区。大力推动马产业发展。加快发展林产业，建设百万亩榛子林基地，实施种苗繁育和苗圃建设项目，发展中草药种业，开发食用菌、浆果等特色产品，做大林业经济。

做大做强龙头企业。以龙头带基地、强品牌、促联结，带动农牧民增收。积极引进伊利等有实力的龙头企业，支持阜丰、晟通、塞尚雀巢、呼伦贝尔肉业集团等龙头企业发展壮大。推动农垦集团释放体制机制活力，成为促进三产融合、现代农牧业发展的引领示范。引导林业集团转变发展方式，促进林业资源保护和开发利用，实现可持续发展。大力实施品牌提升行动，用好“呼伦贝尔”金字招牌和内蒙古味道、呼伦贝尔美食节、北京农超对接等平台，加大呼伦贝尔草原羊肉、牛肉、牛奶、黑木耳等区域公用品牌的培育打造力度，加强品牌营销，扩大宣传影响。完善市本级追溯平台建设，全面推进农畜产品质量安全追溯体系建设，确保农畜产品安全。加快呼伦贝尔现代农牧业产业园、岭东绿色有机食品产业园建设。

加快实施奶业振兴战略。严格落实奶业振兴规划，优化奶业布局，做精做优岭西草原奶业，做大做强岭东半农半牧高效集约奶业发展区。建设优质奶源基地，出台鼓励政策，支持农垦集团在岭东岭西分别建设5000头以上规模养殖场、伊利在阿荣旗建设1.2万头奶牛养殖基地和赛优乳业新增5000头养殖基地建设；支持发展适度规模的奶牛养殖场，大力发展家庭牧场，今年新增和改造升级10个奶牛规模养殖场。强化饲草饲料供给，种植高产苜蓿5万亩以上、粮饲兼用玉米110万亩以上。以打造全国高端液态奶生产基地、优质干乳制品和特色民族奶制品重要产区为目标，鼓励和支持企业加大技术改造和产品研发力度，开发优质干乳制品和高端液态奶产品，确保塞尚雀巢高端乳制品生产线投产。强化奶业安全监管，开展生鲜乳专项整治行动，确保奶源质量安全。

持续打造农村牧区宜居环境。学习借鉴浙江“千村示范、万村整治”经验，坚持典型示范，开展“十县百乡千村”农村牧区人居环境整治示范行动，打造示范旗1个、示范苏木乡镇12个、示范嘎查村100个。以治理“脏乱差”为重点，大力推进农村牧区“厕所革命”、生活垃圾、污水专项治理行动，卫生厕所普及率达到50%以上，有条件的地区生活垃圾得到治理的嘎查村达到50%以上，农村牧区人居环境整治的村庄达到80%以上。全面清理整治“大棚房”，坚决遏制农地非农化。

（五）坚持调存量、优增量，努力加快新旧动能转换。坚持改造与培育并举，加快改变“四多四少”状况，着力振兴工业经济。

全力释放传统产业活力。加快传统产业提质升级，重塑价值链、产业链、供应链，加速资源型产业改造提升。推进金新化工、驰宏冶炼、中国黄金、国森矿业等企业技术改造和产业链延伸，推动产品向高端化、终端化迈进。引导阜丰集团强链增链补链，全力推动50万吨氨基酸及配套项目前期工作。确保沃丰公司年产10万吨液体肥、30万吨生态肥项目开工。加快推动满洲里木材产业向规模化和精深加工转变。

全力加快新兴产业培育。积极推动新能源、新材料、节能环保、高端装备、云计算、生物科技、临空产业、蒙中医药等战略性新兴产业发展，促进项目落地，完善产业链条。加快海拉尔海国华创大数据中心一期、牙克石工业产品耐冷性检验检测大数据、陈旗冬季汽车试验试驾基地、阿荣旗和莫旗生物质发电以及牧业机械生产线智能化改造、绿色再生能源基地等项目建设，确保阿荣旗齐鲁生物制药项目上半年开工。以扎兰屯内蒙古飞行学院为依托，发展飞机维修、航空物流、特色旅游等相关产业，加快推进临空产业发展。

全力推动工业园区振兴。着力解决园区大杂烩、空心化、同质化问题。完善各级工业园区总体规划，明确功能定位，优化产业布局，发挥园区集聚效应。完善基础设施、环保设施和服务功能，提升园区吸引力、集聚力、辐射力。加快盘活和清理园区僵尸企业。加大精准招商力度，尽快落地一批符合高质量发展要求的企业和项目，促进主导产业集中、优势产业集聚。深化与清华大学生态文明研究中心合作，加快编制和实施经济技术开发区产业发展规划，让经济技术开发区焕发新的生机，成为“产业生态化、生态产业化”示范园区。

（六）坚持提品质、促融合，推动服务业提质增效。聚焦品质提升，坚持重点突破，发展新业态，培育新热点，加快旅游业高质量发展，促进服务业全面升级。

推动旅游业提档升级。深度挖掘、整体提升优质旅游资源利用水平，全力打造国际化高端旅游目的地。编制《呼伦贝尔全域旅游发展总体规划》，高标准创建全域旅游示范区。加快呼伦贝尔大草原旅游区、额尔古纳湿地景区、柴河景区5A级创建。提升蒙古之源·蒙兀室韦民族文化园、鲜卑历史文化园等景区运营水平。推进旅游、生态、民族、文博、民俗深度融合，创新打造一批高端旅游产品，加快构建观光旅游、自然教育、休闲体验、康养度假多业态竞相发展的新格局，确保森林草原旅游列车开通。借助“十四冬”，打好冰雪牌，加快建设冰雪运动名城，提升“冰雪那达慕”“中俄蒙国际冰雪节”“中国冷极节”等冬季旅游活动影响力，培育打造“冬季英雄会”、冬季马拉松等品牌活动，以冰雪运动、品牌活动带动冰雪产业发展，把“冷资源”做成“热产业”。加强与蒙东、东北三省和俄蒙毗邻地区旅游合作，深化“4+1”城际旅游联盟合作内涵，加快推进“乌阿海满”旅游一体化发展。以品质提升为重点，补齐基础设施短板，加大重点旅游地区污水、垃圾处理设施建设力度，完善旅游驿站、信息服务平台等配套设施，提升宾馆酒店服务质量和水平，增强游客体验感和满意度。深入开展旅游秩序整治专项行动，加强景区和旅游企业日常监管，提高旅游市场综合执法水平。强化源头管控，继续推进“小散乱”景区景点清理整顿，保护好原生态旅游资源。

加快现代服务业发展。优化商贸物流布局，建设岭东、岭西、岭上区域物流中心，全力打造内通东北、外联俄蒙的区域物流节点地区。加快发展电商物流，规范发展冷链物流，建设信息化、便捷化的电子商务物流体系。大力开展“互联网+”行动，加快电商线上线下融合发展，抓好电商产业园和创业孵化基地建设，形成扎兰屯、阿荣旗、鄂伦春旗、莫旗和其他旗市区梯次发展、各具特色、服务完善的电商发展格局。积极发展文化产业，大力开发文化资源，加快文化产业高质量发展。积极推动现代金融、健康养老、家政服务、会展服务、信息咨询等新兴服务业发展，促进服务业效益提升。启动建设呼伦贝尔国际会展中心。

（七）坚持补短板、强基础，大力改善城乡发展条件。坚持城乡统筹，完善功能，加强基础设施建设，加快产城融合发展，不断提高承载能力，夯实发展基础。

加快宜居城镇建设。优先发展中心城区，坚持新城建设和老城改造并举，提高中心城区、城关镇、特色小镇的承载能力。以承办“十四冬”为契机，加快推进海拉尔区、牙克石市、扎兰屯市城市改造提升，提高绿化、美化、亮化水平。推进智慧城市、海绵城市建设，大力实施供水、供热、供气、排水等市政项目，加快实施中心城区配水管网改造、供热保障及互联互通工程，全面提升城市基础设施保障能力。完善停车场、水冲式公共厕所、城市标识等配套设施，提升城市品位。加快莫尔道嘎、柴河全国特色小镇建设，发展壮大一批产业突出、规模适度、具有带动力的综合型强镇。

提升城市管理水平。深入推动全国文明城、国家卫生城创建，整合城市管理资源，建立三级管理体制，健全标准体系，完善精细化、网格化管理服务机制，做到建管并重、建管衔接、以管促建。深入开展“城市精细化管理年”“物业管理服务质量提升年”活动，以“三清”治理为重点，大力开展综合整治行动，解决脏乱差、交通拥堵、停车难等城市病，打造优良人居环境。

加强基础设施建设。计划投资52亿元，实施33个重点项目。加快推进海拉尔—满洲里、尼尔基—腾克、库布春林场—根河等续建和紫沟—大兴、根河—牙克石等新建公路项目建设。全力支持林区公路建设。全力推动阿荣旗—莫旗铁路项目，确保阜丰铁路专用线开工。积极推进齐海满高铁列入国家“十四五”铁路发展规划，启动海拉尔—伊敏—阿尔山铁路升级改造前期工作。完成海拉尔机场平行滑行道建设和T1航站楼及附属设施扩建工程，加快推进室韦通用机场前期工作，推动满归机场试飞，阿荣旗、莫旗机场通航，基本形成支线与通用航空相互结合的航空网络。继续实施甘河、根河、音河、诺敏河治理工程，加快扎敦水利枢纽工程建设。加快推进扎兰屯市和鄂伦春旗大杨树镇热电联产项目建设。实施海拉尔—满洲里500千伏输变电工程等电网项目。加快推进中俄东线天然气管道呼伦贝尔支线工程项目。

（八）坚持增活力、强动力，深化改革扩大开放。不断破解发展瓶颈，拓宽发展空间，集聚发展要素，让改革开放红利充分释放。

抓好重点领域改革。深化“放管服”改革，最大限度激发市场活力。全面实行“一网一门一次”改革，大力推进“互联网+政务服务”，政务服务事项网上可办率不低于70%。深入推进商事制度改革，落实“多证合一”“证照分离”改革，强化“双随机一公开”监管，完善信用体系建设。建立营商环境评价机制，畅通群众监督渠道，继续整治痛点、难点、堵点，努力增强群众的获得感、满意度。扎实做好农村牧区集体产权制度、土地确权、“三权”分置、农企利益联结四项改革。推进市本级国有资产集中统一监管，提高国有资产配置效率，实现国有资产保值增值。全面实施事业单位公车改革。

促进民营经济发展。制定促进民营经济高质量发展具体举措。落实支持民营经济发展各项政策，着力减轻企业税费负担。建立重点项目融资对接机制，促进金企有效对接。加大金融支持力度，降低企业生产成本和制度交易成本，破解民营企业发展难题。深入落实推进企业上市挂牌三年实施计划，加大企业上市培育力度，增强企业融资能力。营造公平竞争环境，拓宽民营经济发展空间，支持民营企业投资现代农牧业，鼓励民间资本进入教育、医疗、养老、文化、体育等领域，坚决有力支持民营企业发展壮大。

深入实施创新驱动。落实人才引进培育各项政策，切实加强人才工作。全面落实鼓励企业创新各项政策，加速企业技术研发和科技创新步伐。深化科技体制机制改革，突出科技支撑，提供全方位创新创业服务，建设一批“双创”示范平台。加快培育技术创新战略联盟，建设绿色农业产业技术转移转化公共平台。落实与中国农科院战略合作框架协议，组建绿色发展智库、智慧牧业院士专家工作站，共建技术转移转化平台和中试基地。推进呼伦贝尔农垦集团、内蒙古蒙草集团与中科院、深圳华大基因研究所合作，共建国家基因库呼伦贝尔高寒种质资源库。

全力推进对外开放。积极融入“一带一路”建设，以中俄、中蒙建交70周年为契机，深化同俄蒙毗邻地区务实合作。全力推动呼伦贝尔中俄蒙合作先导区和满洲里国家重点开发开放试验区建设，发挥满洲里向北开放的“龙头”作用，支持满洲里综合保税区、平行进口汽车试点口岸、边境旅游试验区建设。深入落实边境口岸建设发展指导意见，全力打造分工明确、重点突出、优势互补的口岸集群。加快推进铁路、公路、跨境桥梁、航空口岸建设，提高互联互通水平。推动边民互市贸易政策覆盖全市、入境的俄蒙8座以下小汽车延伸到呼伦贝尔全域，争取粮食进口指定口岸获批，加快进口粮油加工和食用菌出口基地建设，切实促进开放型经济深入拓展。办好海拉尔第十五届中俄蒙经贸展洽会。大力发展服务贸易、物流业、跨境电子商务和跨境旅游业，加速“通道经济”向“落地经济”转变。加快通关一体化进程，提高对外开放管理服务能力。积极开展政府互访、民间互动，促进与俄蒙毗邻地区在经贸、旅游、文化、教育、体育、科技、医疗、生态等领域合作不断取得新成果。提升区域合作水平，深化与东北三省沟通对接，加强与京津冀、环渤海、长三角、珠三角地区交流合作，办好上海招商推介会、京呼对口帮扶和产业合作恳谈会，实现区域间合作共赢。

（九）坚持保基本、兜底线，着力保障和改善民生。紧扣群众期盼，坚持问题导向，完善制度，守住底线，倾情倾力改善群众生活，让各族群众有更多更直接的获得感、幸福感、安全感。

着力提升保障水平。深入推进“大众创业、万众创新”，加强创业园、孵化基地建设，强化就业创业培训，促进多渠道就业，做好高校毕业生、就业困难人员、农牧民工等重点群体就业，全年城镇新增就业2.3万人，城镇调查失业率、登记失业率分别控制在5.5%左右和4.5%以内。继续实施保障性安居工程，实施城市棚户区改造1400套，完成农村牧区危房改造841户。织牢织密社会保障网，加快推进养老保险制度改革，提高职工基本医疗保险水平。稳步提高城乡低保、社会救助、抚恤优待等标准，探索建立丧葬补助金制度，开展农村牧区低保专项治理，鼓励社会力量开办养老机构。

统筹推进社会事业。坚持教育优先发展，全面落实深化新时代教师队伍建设改革实施意见。实施高中阶段教育普及攻坚计划和第三期学前教育三年行动计划，普惠性幼儿园覆盖率达到75%。完成海二中搬迁，积极推进海一中迁建和新区学校建设。落实高考综合改革政策。加快发展现代职业教育，支持呼伦贝尔学院转型发展，着力提升高等教育服务经济社会发展能力。深入推进医药卫生体制改革，全力提高医疗服务水平，建立健全现代医院管理制度，落实医保支付改革政策，加快分级诊疗和医联体建设，扩大优质医疗资源覆盖面。扎实推进文化事业繁荣发展，大力弘扬“乌兰牧骑精神”，加大文艺创作力度，努力推出精品力作。加强民族文化保护，编制“三少民族”文化保护传承发展规划。实施呼伦贝尔民族博物院和鄂伦春旗博物馆改造、内蒙古蒙古族源博物馆布展、乌兰牧骑宫和新闻中心续建等工程，确保呼伦贝尔历史博物馆开工建设。大力发展体育事业，高质量完成“十四冬”场馆建设，举全市之力做好大型活动、氛围营造、服务保障等各项筹备工作。广泛开展全民健身活动，推进足球运动改革，集中打造曲棍球、冰雪运动等优势体育项目，提升体育事业发展水平。扎实做好第四次全国经济普查。积极支持审计、地震、双拥、气象、老龄、工青妇、红十字、残疾人保障、关心下一代等事业发展。

创新细化社会治理。推动社会治理重心下移，推进基层治理现代化。深化平安呼伦贝尔、法治呼伦贝尔建设，深入推进扫黑除恶专项斗争，严厉打击各类违法犯罪活动。牢固树立安全发展理念，强化安全隐患排查整治，坚决遏制重特大事故发生。加强防灾减灾和应急保障体系建设，提升突发事件应急处置能力和保障水平。全力做好森林草原防火工作。加强市场监管，依法打击制假售假侵权等行为。严密防范非洲猪瘟疫情。深入推进国家食品安全示范市创建。坚持依法治访，推动积案化解和依法分类处理，妥善解决群众合理诉求。强化社区治理能力，做好法律援助、社区矫正、人民调解、安置帮教等工作。加强国防动员工作，加大管边控边力度，提升边境综合防控能力，促进军民融合发展。扎实推进民族团结进步事业，加快“三少民族”自治旗发展，维护民族团结、边疆安宁、和谐稳定的良好局面。

（十）坚持转作风、强素质，切实加强政府自身建设。牢记群众期盼，对人民负责，受人民监督，让人民满意。

加强政治建设，做到绝对忠诚。坚持用习近平新时代中国特色社会主义思想武装头脑、指导实践、推动工作。树牢“四个意识”，坚定“四个自信”，坚决做到“两个维护”，牢记为民宗旨，严守政治纪律，始终在思想上政治上行动上同以习近平同志为核心的党中央保持高度一致，坚决有力、不折不扣地把中央决策部署和自治区、市委工作部署落到实处。

加强能力建设，做到担当尽责。全面加强学习，改进调查研究，坚持深入基层、深入群众，掌握实情、善谋实事，不断增强履职本领，提高科学决策水平和解决问题能力。树立苦干实干导向，坚决整治“四种行为”“四官”问题，勇于拼搏进取，善于攻坚克难，推动各项工作部署落地落实。全力推进政府机构改革，加快政府职能转变，提高行政效能，打造高效便捷、公开透明政务环境。

加强法治建设，做到依法行政。建设法治政府，把政府工作全面纳入法治轨道。坚持依法决策、民主决策、科学决策，明确法定职责，严格权力边界，自觉按法定权限和程序履行职责、行使职权。落实政府法律顾问制度。依法接受人大法律监督和工作监督，自觉接受政协民主监督，自觉接受监察机关监督，主动接受社会监督，加强审计监督，让权力在阳光下运行。

加强廉政建设，做到严守底线。推进“两学一做”学习教育常态化制度化，深入开展“不忘初心、牢记使命”主题教育。认真落实全面从严治党主体责任，严格执行中央八项规定及其实施细则，驰而不息正风肃纪，把从严治党责任落实到从严治政全过程。坚持用制度管权管事管人，加强对重点领域、关键岗位的监督。强化廉政教育，坚决做到清廉从政，干净做事，以为民务实清廉的形象取信于民。

各位代表，实干成就梦想，奋斗铸就辉煌。今天的呼伦贝尔已经站在高质量发展的新起点上，让我们在自治区党委、政府和市委的坚强领导下，以习近平新时代中国特色社会主义思想为指导，紧紧依靠和团结全市各族人民，凝心聚力，埋头苦干，不断开创经济社会发展新局面，为建设美丽富饶和谐安宁的呼伦贝尔而不懈奋斗，以优异成绩庆祝中华人民共和国成立70周年！
